# Robust Face Recognition Based on a New Supervised Kernel Subspace Learning Method

**DOI:** 10.3390/s19071643

**Published:** 2019-04-06

**Authors:** Ali Khalili Mobarakeh, Juan Antonio Cabrera Carrillo, Juan Jesús Castillo Aguilar

**Affiliations:** Department of Mechanical Engineering, University of Málaga, Doctor Ortiz Ramos s/n, 29071 Malaga, Spain; jcabrera@uma.es (J.A.C.C.); juancas@uma.es (J.J.C.A.)

**Keywords:** biometrics, dimensionality reduction, face recognition, kernel trick, subspace learning, manifold learning

## Abstract

Face recognition is one of the most popular techniques to achieve the goal of figuring out the identity of a person. This study has been conducted to develop a new non-linear subspace learning method named “supervised kernel locality-based discriminant neighborhood embedding,” which performs data classification by learning an optimum embedded subspace from a principal high dimensional space. In this approach, not only nonlinear and complex variation of face images is effectively represented using nonlinear kernel mapping, but local structure information of data from the same class and discriminant information from distinct classes are also simultaneously preserved to further improve final classification performance. Moreover, in order to evaluate the robustness of the proposed method, it was compared with several well-known pattern recognition methods through comprehensive experiments with six publicly accessible datasets. Experiment results reveal that our method consistently outperforms its competitors, which demonstrates strong potential to be implemented in many real-world systems.

## 1. Introduction

In the field of face recognition, many different dimensional recognition approaches have been developed in recent times [1,2,3]. Dimensionality reduction is a main problem in numerous recognition techniques, caused by the great amount of data with high dimensions in many real-world utilizations [4,5,6]. In fact, dimensionality reduction techniques have been recommended by researchers to avoid “the cure of dimensionality,” so as to amend the computational efficiency of image recognition [7,8]. Generally, dimensionality reduction techniques can be classified into two main groups: i.e., linear and nonlinear. In linear methods, a significant low-dimensional subspace is intended to be discovered in the input data with high-dimensional space, where the embedded data in the input space has a linear structure [9,10,11,12]. Principle component analysis (PCA) is one of the most famous linear methods [13,14,15]. PCA aims to retain global geometric information for data representation through enhancing the trace of the feature covariance matrix [13,16,17]. Linear discriminant analysis (LDA) is a linear technique that seeks to find out the discriminant information for data classification by enhancing the ratio between inter-class and intra-class scatters [16,18]. Some of the limitations of both PCA and LDA are that they could suffer from the small sample size (SSS) [15] issue, and that they may fail to recognize many important data structures that are nonlinear [19,20].

Scholars have developed abundant practical nonlinear dimensionality reduction strategies [21] to address these problems. They can be classified into two types: manifold-learning-based and kernel-based techniques [22,23]. Manifold learning directly aims to discover principal nonlinear data with low-dimensional structures that are concealed in the input space. Isometric feature mapping (ISOMAP) [24,25] and local linear embedding (LLE) [26,27] are the most well-known manifold-learning-based techniques to find inherent low-dimensional embedding of data [28]. Based on some experiments carried out using these techniques, it is illustrated that these methods can discover meaningful embedded nonlinear data structures for face images adequately.

However, manifold-learning-based techniques could suffer from two issues in terms of pattern recognition [15]. The first one is called “overlearning of locality,” [29] since manifold learning keeps locality data structures but there is no straight connection with classification. Out-of-sample is another issue that shows why most manifold-learning-based techniques are not appropriate for image recognition tasks [30,31]. These techniques can yield an embedding directly from a training data set, but they are often not able to find the sample’s image in the embedding space when it is implemented to a new point. These problems cannot be overcome by the currently proposed manifold learning methods. Although a few supervised forms have been proposed, they still suffer from these problems [30,31,32] because they are all founded based on “locality” characterization. The local quantity is sufficient for one manifold modeling, but it does not work well for classification tasks in multi-manifold modeling [17].

In contrast to manifold-learning-based techniques and in order to indirectly represent observed patterns into possibly much larger dimensional feature vectors, kernel-based techniques have been proposed by applying a kernelized nonlinear representation method. In this approach, the nonlinear data structure can be more separable in the observation space and become linear in the feature space. The representative strategies include kernel Fisher discriminant (KFD) [23,33] and kernel principal component analysis (KPCA) [34,35]. Both have shown that they can be practical in many real-world functions, such as face recognition, to preserve the nonlinear data structure [36]. However, these kernel-based methods cannot directly consider the local data structure, which results in classification performance degradation.

Recently, the locality preserving projections (LPP) method has been proposed as a linear subspace learning method to address the out-of-sample problem [37]. LPP is an unsupervised linear subspace technique that has the remarkable advantage of being able to generate an explicit map. Similar to the one belonging to PCA and LDA, this map is linear and easy to compute, and is also effective for many face recognition tasks. Although LPP is designed based on “locality,” like most manifold learning methods, it still suffers from the “over learning of locality” problem, because there is no direct connection with classification in its algorithm. Therefore, on some occasions, it cannot be guaranteed to map an appropriate projection for classification purposes [37].

Subsequently, in order to address this issue and delve into more influential projections for classification tasks, the unsupervised discriminant projection (UDP) method was developed as a simple version of LPP [38]. UDP is considered a linear estimation of multi-manifold-based learning because it considers both the local and nonlocal scatters of data. In both LPP and UDP, the data class label information is not considered, which may degrade their pattern classification performance. Furthermore, the discriminant neighborhood embedding (DNE) method has been presented with the idea of using data class label information [39]. DNE can find a good embedding for classification, considering intra-class absorption and inter-class class expulsion. The main characteristic of DNE is called “discrimination,” meaning the ability to distinguish the same class from distinct classes. This specification of DNE can deal well with “out-of-sample” and “small training sample size” problems. Nevertheless, DNE cannot correctly preserve local information of data because it only concedes +1 and −1 to intra-class and inter-class neighbors [15]. Thus, much of the important geometrical structure information of data may be lost, and it might fail to find out the most significant sub-manifolds for pattern recognition. The locality-based discriminant neighborhood embedding method (LDNE) has recently been proposed to tackle the problems existing in LPP and DNE [15]. This method takes into account both the “locality” and the “discrimination” in a united modelling environment. However, many important non-linear data might be lost during the dimensionality reduction process, which dramatically influences classification accuracy.

According to the way dimensionality reduction algorithms “learn” from data to create predictions, they can be categorized into two different classes: supervised and unsupervised learning methods. Among these two, supervised machine learning is used more prevalently. In this case, the data scholar acts as a guide to instruct the algorithm regarding which results should be found by it [40]. The most well-known supervised algorithms include supervised LPP [39], local discriminant embedding (LDE) [41], neighborhood discriminant projection (NDP) [42], discriminant locality preserving projections (DLPP) [43], locally discriminating projection (LDP) [44], and geometry preserving projections (GPP) [45] It is clear that the aforementioned supervised techniques generally apply class label information in order to amend the dimensionality reduction. On the other hand, the unsupervised LPP-based algorithms generally aim to improve the locality preserving and discriminating capabilities to further enhance the final performance of the classification process. Graph-optimized locality preserving projections (GOLPP) [15,46], orthogonal locality preserving projection (OLPP) [47], and UDP [38] are some examples of the unsupervised LPP-based methods.

In this project, a new supervised subspace learning algorithm named “supervised kernel locality-based discriminant neighborhood embedding” (SKLDNE) is proposed. In this approach, not only can the nonlinear data structure be preserved by applying a kernelized nonlinear mapping method, but also both “locality” and “discrimination” of data in an integrated modeling environment are considered simultaneously. It should be noted that this technique is supervised through direct connection with classification in order to properly guide the procedure of dimensionality reduction. In order to have a reliable and powerful comparison, the efficiency of the proposed SKLDNE technique was compared with PCA, KPCA, LDA, UDP, LPP, DNE, and LDNE by a broad range of experiments with different publicly available face datasets, i.e., Yale face, Olivetti Research Laboratory (ORL) face, Head Pose, and Sheffield. Moreover, Finger Vein and Finger Knuckle databases were also applied to investigate the implementation of our algorithm in other types of databases rather than faces.

It is worthwhile to highlight several characteristics of the proposed approach here:

(1) SKLDNE has been successfully designed to retain local geometric relations of the within-class samples, which are very important for image recognition. Generally, the categorization strength of methods with a linear learning algorithm is restricted. They fail to deal with complicated problems. Many effective nonlinear data features may be lost during the classification progress using linear techniques such as LDNE, LDA, DNE, and LPP. Therefore, applying a nonlinear method can effectively improve the classification performance.
(2) This technique is a supervised learning method, as the data scholar acts as a guide to instruct the main algorithm whose conclusion should be found. SKLDNE considers class label information of neighbors in which there is a direct connection with classification, in order to enhance final recognition performance.
(3) It benefits from the advantages of “locality” in LPP in which, due to the prior class-label information, geometric relations are preserved.
(4) Not only can it build a compact submanifold by minimizing the distance between the same points in the same class, but it also expands the gaps among submanifolds of distinct classes simultaneously, which is called “discrimination.”
(5) SKLDNE can resolve the SSS problem that is mostly faced by other aforementioned techniques such as PCA, LDA, UDP, and LPP, and the “overlearning of locality” problem in the manifold learning.
(6) Due to its kernel weighting, it is very efficient in reducing the negative influence of the outliers on the projection directions, which effectively handles the drawbacks of linear models and makes it more robust to outliers.

The rest of this study is organized as follows: Section 2 categorizes LPP, DNE, and LDNE. Section 3 is devoted to describing the proposed SKLDNE method and the pertinent algorithm and mathematics. Section 4 focuses on the experiments and analyses carried out. Section 5 elucidates conclusions and future research.

## 2. Outline of LPP, DNE, and LDNE

In this section we will briefly review LPP, DNE, and LDNE, as our proposed method is designed to possess the best characteristics of these techniques in graph embedding.

### 2.1. Locality Preserving Projection

LPP is one of the successful linear algorithms used for dimensionality reduction that finds graph embedding of data sets. The manifold structure is directly modeled through creating the nearest-neighbor graph, disclosing vicinity relations of data points in order to preserve the local structure of the input data in the projection [48]. LPP works based on a linear approximation of a Laplacian Eigen map, which searches a transformation *P* in which input data *X* = [$x_{1}$, $x_{2}$, …, $x_{n}$] with a high dimension is projected into subspace *Z* with a low-dimension, while the local structure is retained [49]. In order to calculate linear transformation T, the objective function should be minimized as follows [43]:

(1) $$\min_{P}\sum_{i,j=1}^{n}{∥z_{i}-z_{j}∥}^{2}H\left(i,j\right)$$

where weight matrix *H* (called heat kernel) is obtained by the nearest-neighbor graph and $z_{i}=P^{T}x_{i}$. If $x_{i}$ is among *l* nearest neighbor of $x_{j}$ or $x_{j}$ is among *l* nearest neighbor of $x_{i}$, then

(2) $$H\left(i,j\right)=e^{-\frac{{∥x_{i}-x_{j}∥}^{2}}{t}}$$

where parameter *t* is an appropriate constant number. Otherwise, H(i,j)=0. On the other hand, when $x_{i}$ and $x_{j}$ are the nearest neighbors, weight matrix *H* could be clearly set as: H(i,j)=1. Otherwise, H(i,j)=0. The optimal transformation matrix can be calculated by converting the minimization problem into solving the generalized eigenvalue problem

(3) $$XLX^{T}P=\lambda XDX^{T}P$$

where *L* = *D* – *H* is the Laplacian matrix and $Dii=\sum_{j}H\left(i,j\right)$ is a diagonal matrix.

### 2.2. Discriminant Neighborhood Embedding

Discriminant neighborhood embedding (DNE) is suggested based on an intuition of a dynamics theory. DNE works under the category of supervised learning, in which multi-class data points are pushed or pulled in a high dimensional space to modulate a favorable embedding of low dimensionality for classification [15]. Furthermore, DNE effectively avoids the complication of a singularity matrix, as there is no need to calculate the inverse matrix. Based on the main characteristics of DNE, it can present a good solution for the small sample size (SSS) and the out-of-sample problems [50]. Although this comprehensive technique is effective in pattern classification, it still cannot uphold the data’s geometrical structure information. The main steps of the DNE algorithm are as follows [39]:

(1) The adjacent matrix $\bar{H}$ of graph G which refers to the underlying supervised manifold structure is as follows:
  (4) $${\bar{H}}_{ij}=\{\begin{array}{c} -1,\;x_{i}\in k\mathrm{nn}\left(j\right)\;\mathrm{or}\;x_{j}\in k\mathrm{nn}\left(i\right)\;\mathrm{and}\;\left(c_{i}\neq c_{j}\right)\\ +1,\;x_{i}\in k\mathrm{nn}\left(j\right)\;\mathrm{or}\;x_{j}\in k\mathrm{nn}\left(i\right)\;\mathrm{and}\;\left(c_{i}=c_{j}\right)\\ 0,\;\mathrm{otherwise} \end{array}$$
  where *c_i_* is the class label of *x_i_* and knn (*i*) is the set of *k* nearest neighbors of *x_i_*. Note that each edge is weighed +1 or −1 respectively in order to determine the local intra-class attraction and inter-class repulsion between neighboring points.
(2) The optimal transformation of matrix *P* can be defined as follows:
  (5) $$\min\sum_{ij}{∥z_{i}-z_{j}∥}^{2}{\bar{H}}_{ij}$$

The minimization problem can be reduced to:

(6) $$\text{argmin tr}\left(P^{T}X\bar{L}X^{T}P\right)$$

Subject to $P^{T}P=I$, where $\bar{L}=\bar{D}-\bar{H}$ and $\bar{D_{ii}}=\sum_{j}\bar{H_{ij}}$ is a diagonal matrix.

Like LPP, parameter *P* (projection matrix) can be optimized by calculating the minimum eigenvalue solution to the generalized Eigen value problem as follows:

(7) $$X\bar{L}X^{T}P=\lambda P$$

where *P* is constituted by the *r* eigenvectors corresponding to its first smallest negative eigenvalues of *d*, i.e., $\lambda_{1}$ ≤ $\lambda_{2}$ ≤…≤ $\lambda_{d}$ < 0 ≤ $\lambda_{d+1}$.

### 2.3. Locality-Based Discriminant Neighborhood Embedding

**Figure 1:**
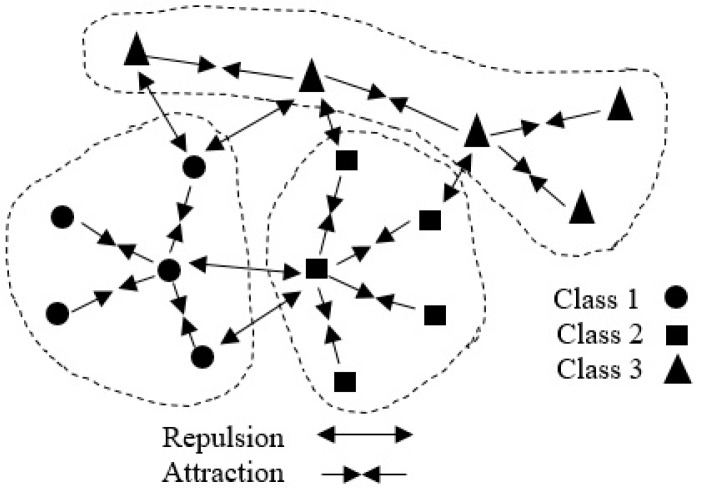
The interactions by attraction and repulsion for the points between different classes.

In the multi-class classification assignment, *N* data points should be classified. The problem that arises here is finding a circumlocutory manifold embedded subspace. Based on the DNE, there are two classifications for the important characteristic of the manifold structure, namely inter-class expulsion, which is the interaction between pairs of neighbors from the same class, and intra-class absorption, meaning the interaction between pairs of neighbors from different classes [15]. These two classes can be defined as follows (Figure 1) [15]:

(1) Intra-class absorption: the interaction between pairs of neighbors from the same class.
(2) Inter-class expulsion: the interaction between pairs of neighbors from different classes.

It is possible to classify all data points based on absorption interaction or distracting behavior using these two classes. Therefore, in the subspace, neighbors from a similar class are absorbed, whereas neighbors from a distinct class become detachable. In order to formulate the method, first we consider that $x_{i}$ is a data point, $N^{s}\left(x_{i}\right)$ is the intra-class neighbors of $x_{i},$ $N^{d}\left(x_{i}\right)$ denotes the inter-class neighbor of $x_{i}$, and $N\left(x_{i}\right)$ represents all the neighbors of $x_{i}$. Thus, to actualize this task with the purpose of understanding these two classes, edges between $x_{i}$ and inter-class neighbors and intra-class neighbors are indicated using various weights. Denoted weights are calculated using a kernel function based on the disparity between $x_{i}$ and its nearby neighbors. Diacritical adjacent graph *D* can be obtained by the ϵ-neighborhood. The diacritical adjacency weight matrix (DAWM) *F* is described as [15,20]:

(8) $$F_{ij}=\{\begin{array}{c} -\exp\left(-\frac{∥{x_{i}-x_{j}∥}^{2}}{t}\right),\;x_{i}\in N_{k}^{s}\left(x_{i}\right)\;\mathrm{or}\;x_{i}\in N_{k}^{s}\left(x_{j}\right)\\ +\exp\left(-\frac{{∥x_{i}-x_{j}∥}^{2}}{t}\right),\;x_{j}\in N_{k}^{d}\left(x_{i}\right)\;\mathrm{or}\;x_{i}\in N_{k}^{d}\left(x_{j}\right)\\ 0,\;\mathrm{otherwise} \end{array}$$

where *t* is the regulator, $N_{k}^{s}\left(x_{i}\right)$ is the intra-class neighbors of $x_{i}$, and $N_{k}^{d}\left(x_{i}\right)$ is the inter-class neighbors of $x_{i}$ in a *k*-neighborhood. It is obvious that different samples lead to different classification results. In view of the fact that the individual feature space location of a sample indicates its conditions, a parameter has been defined to regulate adjacent weight between pairs of neighbors. This regulator can be formulated as:

(9) $$t=\frac{1}{k}\Sigma_{j=1}^{k}{∥x_{i}-x_{j}∥}^{2}$$

To gain intra-class absorption and inter-class expulsion in the transformed space, it is recommended to apply a linear mapping method to project the intra-class absorption and inter-class abhorrence of input data points. As a result, the new low dimensional space can be defined as:

(1) Intra-class absorption:
  (10) $$\begin{array}{c} \Phi \left(P\right)=\Sigma_{ij}{∥y_{i}-y_{j}∥}^{2}W_{ij}=\Sigma_{ij}{∥P^{T}x_{i}-P^{T}x_{j}∥}^{2}W_{ij},\\ x_{j}\in N_{k}\left(x_{i}\right)\;\mathrm{or}\;x_{i}\in N_{k}\left(x_{j}\right)\;\mathrm{and}\;\left(c_{i}=c_{j}\right) \end{array}$$
(2) Inter-class expulsion:
  (11) $$\Psi \left(P\right)=\Sigma_{ij}{∥y_{i}-y_{j}∥}^{2}W_{ij}=\Sigma_{ij}{∥P^{T}x_{i}-P^{T}x_{j}∥}^{2}W_{ij},$$
  $$x_{j}\in N_{k}\left(x_{i}\right)\;\mathrm{or}\;x_{i}\in N_{k}\;\mathrm{and}\;\left(c_{i}\neq c_{j}\right)$$

Finally, the difference between weighted distancse from each data point to the inter-class neighbors in $N^{d}\left(x_{i}\right)$ and those from $x_{i}$ to the intra-class neighbors in $N^{s}\left(x_{i}\right)$ in the mapped space must be calculated and, by maximizing this measurement, we can obtain the optimum result. This measurement can be referred to as a margin, which is calculated as follows:

(12) Θ(P)=Ψ(P)−Φ(P)

Thus, if the original data points are close together, this margin can keep the projected data points as close as possible. However, we can prevent $x_{i}$ and $x_{j}$ from being mapped far apart if they are close by defending the retribution generation [15]:

(13) $$\Theta \left(P\right)=\Sigma_{ij}{∥P^{T}x_{i}-P^{T}x_{j}∥}^{2}F_{ij}$$

## 3. Supervised Kernel Locality-Based Discriminant Neighborhood Embedding

### 3.1. Main Idea

As already mentioned in Section 1, DNE cannot correctly preserve local information of data because it only concedes +1 to intra-class and −1 to inter-class neighbors, so it might fail to find out the most significant submanifold for pattern classification. In addition, LPP is designed based on “locality” since it has no direct connection with classification, and it still suffers from the “over learning of locality” problem [17]. Therefore, LDNE has been proposed to overcome the problems existing in LPP and DNE, considering both the “locality” and the “discrimination” in a unified modeling setting. However, it does not guarantee an appropriate projection for classification purposes, because many important non-linear data might be lost during its dimensionality reduction process. In some cases, LDNE also cannot distinguish inter-class and intra-class neighbors properly in order to conduct projection for all points, which can degrade the classification performance. In order to address these problems, we propose a new supervised subspace learning method named “supervised kernel locality-based discriminant neighborhood embedding” (SKLDNE). Combined with nonlinear data structure, locality and discrimination information, SKLDNE can yield an optimal subspace that best finds the indispensable submanifolds-based structure.

In our proposed SKLDNE, we first use nonlinear kernel mapping to represent the input data in implied feature space *F*. Afterwards, a linear transformation is searched to retain within-class geometric structures in the feature space. Hence, we can achieve a nonlinear subspace that is able to estimate the essential geometric structure of the face manifold. As a matter of fact, the proposed SKLDNE is modeled to take the nonlinear data in the feature space while important features of data including “locality” and “discrimination” are simultaneously preserved. In order to clearly elucidate the performance of our SKLDNE, we have compared it with several dimensionality reduction techniques including PCA, KPCA, LDA, UDP, LPP, DNE, and LDNE on six different publicly available datasets.

### 3.2. Mathematics

Suppose *X* = [$x_{1},\;x_{2}$, …, $x_{n}$] is a set of d-dimensional input samples. This input data is projected onto a higher dimensional feature space *F* via nonlinear mapping Ø: $R^{n}$→*Ϝ*. Then, manifold learning is then carried out on the projected samples $\emptyset \left(X\right)=[\emptyset (x_{1}),\emptyset (x_{2}),\ldots ,\emptyset (x_{n})]$. Now assume that we have to find a projection transformation $V_{\emptyset }$ in *Ϝ*. The optimization problem can be expressed as:

(14) $$\mathrm{Maximize}\;\sum_{ij}^{n}{∥z_{i}-z_{j}∥}^{2}F_{ij}$$

Subject to ${V_{\emptyset }}^{T}V_{\emptyset }=I$, where *I* denotes the identity matrix, $z_{i}=v_{\emptyset }^{T}\emptyset (x_{i})$ and $z_{j}=v_{\emptyset }^{T}\emptyset (x_{j})$ are the projection of $\emptyset (x_{i})$ and $\emptyset (x_{j})$ respectively onto $V_{\emptyset }$, and $F_{ij}$ represents the relationship between of $x_{i}$ and $x_{j}$. The optimization problem can be kernelized as:

(15) $$\sum_{ij}^{n}{∥z_{i}-z_{j}∥}^{2}F_{ij}=\sum_{ij}^{n}{∥v_{\emptyset }^{T}\emptyset (x_{i})-v_{\emptyset }^{T}\emptyset (x_{j})∥}^{2}F_{ij}$$

This equation can be rewritten from the square of norm in Equation (15) into the form of trace as follows:

(16) $$\begin{array}{l} \sum_{ij}^{n}{∥v_{\emptyset }^{T}\emptyset (x_{i})-v_{\emptyset }^{T}\emptyset (x_{j})∥}^{2}F_{ij}\\ \;=\mathrm{tr}\;\left\{\sum_{ij}^{n}\left(v_{\emptyset }^{T}\emptyset (x_{i}\right)-v_{\emptyset }^{T}\emptyset (x_{j}))\left(v_{\emptyset }^{T}\emptyset (x_{i}\right)-v_{\emptyset }^{T}\emptyset (x_{j}){)}^{T}F_{ij}\right\}\\ \;=\mathrm{tr}\;\{v_{\emptyset }^{T}\sum_{ij}^{n}\left(2\emptyset (x_{i}\right)F_{ij}\emptyset {\left(x_{j}\right)}^{T}-\left(2\emptyset (x_{j}\right)F_{ij}\emptyset {\left(x_{i}\right)}^{T}\;F_{ij})V_{\emptyset }\} \end{array}$$

The linear transformation should lie in the span of $\emptyset (x_{1}),\emptyset (x_{2}),\ldots ,\emptyset (x_{n})$ and $\alpha =\left[\alpha_{1},\alpha_{2},\ldots ,\;\alpha_{n}\right]$ are the expansion coefficient vectors and

(17) $$V_{\emptyset }=\sum_{i=1}^{n}\alpha_{i}\emptyset (x_{i})=\emptyset \left(X\right)\;\alpha$$

Substituting (17) into (16) we obtain:

(18) $$\begin{array}{c} U_{\emptyset }=2\mathrm{tr}\;\left\{v_{\emptyset }^{T}\emptyset \left(X\right)\left(D-F\right)\emptyset {\left(X\right)}^{T}V_{\emptyset }\right\}=2\mathrm{tr}\;\left\{v_{\emptyset }^{T}\emptyset \left(X\right)L\;\emptyset {\left(X\right)}^{T}V_{\emptyset }\right\}\\ =2\sum_{l=1}^{m}\{v_{\emptyset l}^{T}\emptyset \left(X\right)L\;\emptyset {\left(X\right)}^{T}V_{\emptyset l}\} \end{array}$$

where $D_{ii}$ = $\sum_{j}F\left(i,j\right)$ is diagonal matrix and *L* = *D* − *F*, *L* and *D* are the symmetric matrix and represent the number of eigenvalues. The optimization problem can be rewritten as:

(19) $$\mathrm{Maximize}\;\mathrm{tr}\;\left\{v_{\emptyset }^{T}\emptyset \left(X\right)L\;\emptyset {\left(X\right)}^{T}V_{\emptyset }\right\}=\alpha^{T}KLK\alpha$$

Subject to $\alpha^{T}K\alpha =I$, where *K* is a kernel matrix with $k\left(xi,xj\right)=[\;\emptyset (x_{i}).\emptyset (x_{j})]$, and a kernel in matrix form is:

(20) $$K=\emptyset {\left(X\right)}^{T}\emptyset \left(X\right)$$

The corresponding generalized eigenvalue problem can be solved through calculating the maximum eigenvalues in $\emptyset \left(X\right)L\;\emptyset {\left(X\right)}^{T}V_{\emptyset }=\lambda V_{\emptyset }$, where the generalized eigenvector equivalent to the biggest eigenvalue is the main interest. Finally, we need to compute the dot product through a kernel and find its nearest neighbor in the embedding space. It should be noted that, although SKLDNE and other methods like DNE consider geometrical information of both intra-class and inter-class, the construction of their contiguous graphs and weights are done in very diverse ways.

## 4. Biometrics Application: Results and Analysis

In this part, to have a reliable and powerful comparison, the implementation of the suggested SKLDNE technique was compared with PCA, KPCA, LDA, UDP, LPP, DNE, and LDNE by broad comparative experiments on six different publicly available datasets, i.e., Sheffield, Yale face, ORL face, Head Pose, Finger Vein, and Finger Knuckle Databases [15]. For each dataset, depending on the number of data for each class, some samples were randomly chosen as training samples, whereas the rest of that class was chosen for testing. Furthermore, in the recognition phase [15], due to simplicity and in order to make the recognition outcome favorable, the nearest neighbor was utilized as a classifier. *K*-neighborhood variable *k* for computing the weight matrix is marked by *Wk* in the posterior explanations. In all the experiments, to achieve fair comparisons, *Wk* was selected as *Wk* = *Tn* − 1 (where *Tn* is training number samples of every class). Based on our experiments, all aforementioned methods achieved the optimal recognition rate with this value of *Wk*. It should be noted that there are two different kernels called Polynomial and Gaussian kernel. Based on our experimental results, the Polynomial has shown better performance than the Gaussian one. Therefore, only the polynomial kernel type was applied in this research.

### 4.1. Experimental Results with the Sheffield Face Database

**Figure 2:**
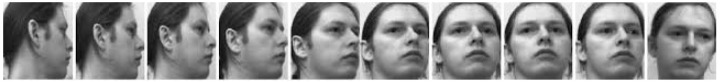
A sample of pre-cropped face images in the Sheffield Face database [51].

The Sheffield Face Database (SH.F) [51] includes a total of 20 individuals, with 564 images in which each subject is illustrated, including poses ranging from profile to forehead views. The format of all images is PGM, image size is approximately 220 × 220 pixels in 256-bit grayscale. Figure 2 illustrates a one-subject sample with different poses from the Sheffield face multi view. In our experimentation, every image was resized to 112 × 92 pixels [52]. The maximal rate of recognition of each method and the related dimension implemented in the Sheffield database are illustrated in Table 1. The best performance result among other methods is assigned in bold-text. In addition, in all experiments due to the large number of implementations, it was decided to select some training and testing images that were more challenging for the classification task, in order to evaluate the performance of each technique in these critical areas. Thus, considering the small training sample size problem (SSS), first we selected a small number of training samples and then some large numbers to evaluate the performance of our SKLDNE method accurately. *Tn* = 1, 2, 3, 4, 5, 6, 7, 8, 15, 16, 17 were the training sample numbers selected from each class.

**Figure 3:**
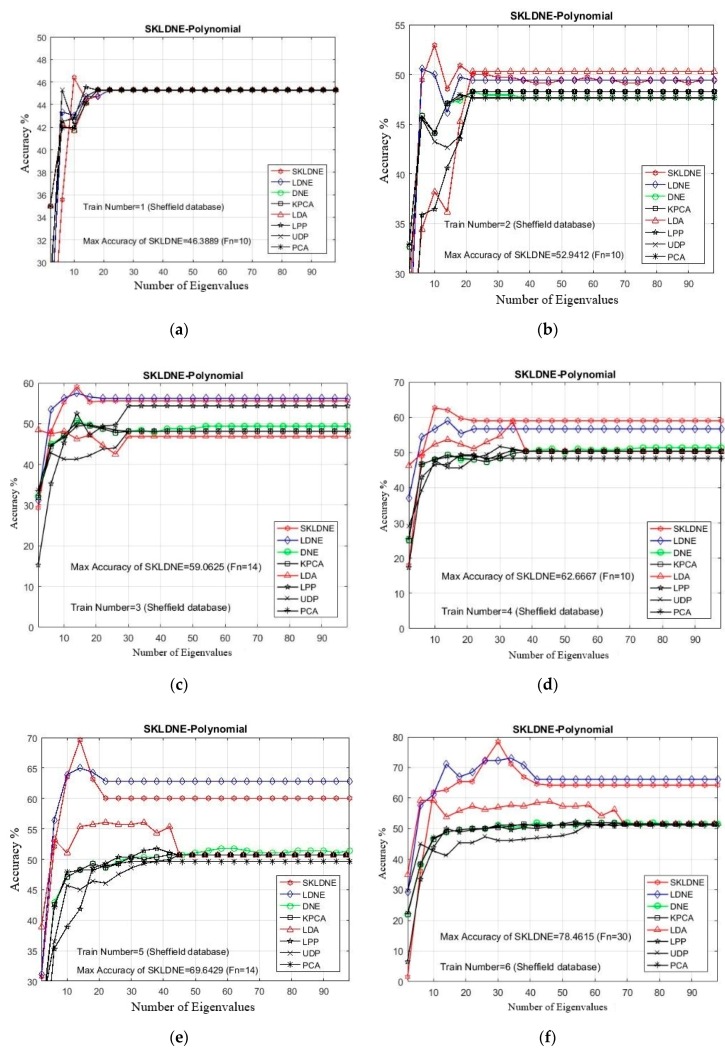

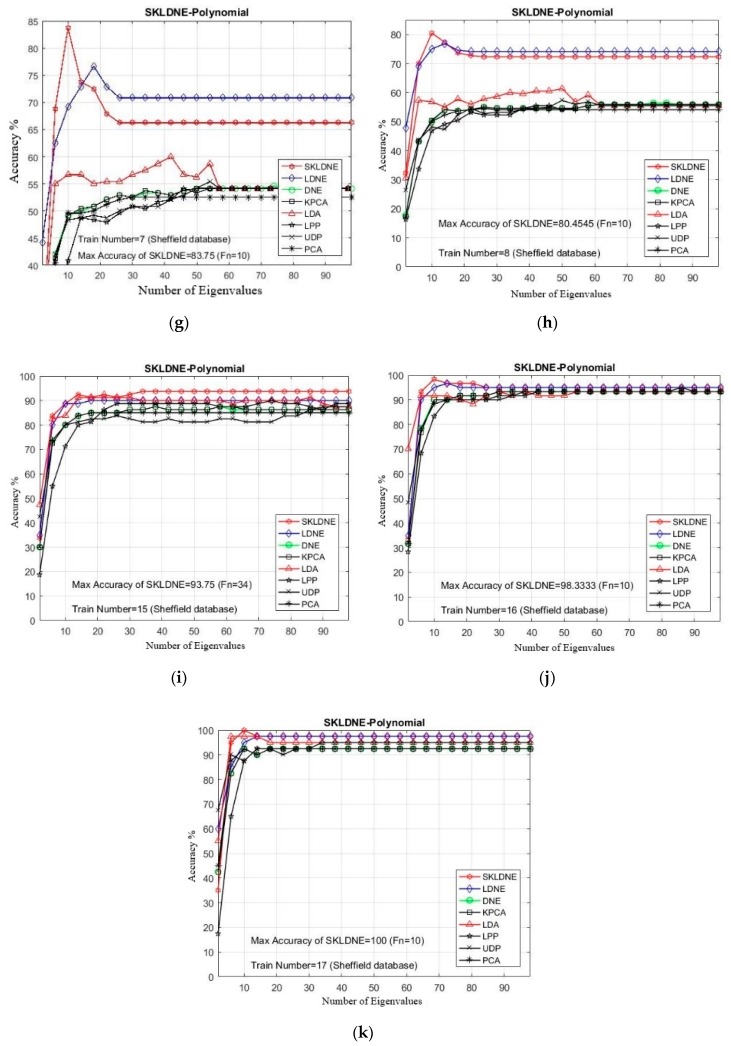
(**a**–**k**) The comparative recognition results changing the dimensionality of the transformation matrix for each given training number Tn on each data (Sheffield Face database).

According to Table 1, three main conclusions can be drawn. First, SKLDNE significantly outperformed other methods (PCA, KPCA, UDP, LPP, LDA, DNE, and LDNE) over an extensive range of dimensionality for all different numbers of training and testing images, whether the training sample size was large or small. As can be observed, when the training sample number was small, SKLDNE clearly behaved more efficiently than all other recognition techniques, which proves the robustness of this method in the case of the small training sample size problem (SSS). Secondly, it can be seen that the recognition rates of all implementations were better when more training samples are used. Third, as shown in Figure 3, SKLDNE provided the best results at the lowest number of dimension values compared to its competitors. In addition, when dimensionality increased to about 30, the recognition accuracy of each method first increased rapidly and then became stable. The differences between the recognition rate of SKLDNE and other methods were emphasized when the training sample number was very small. However, for the larger training sample, the mentioned differences in the classification rate increased. For instance, in the training number of 1, 2, 3, SKLDNE achieved much better results than others. Moreover, for *Tn* = 17, SKLDNE reached 100% recognition rate, while the results were much lower for other methods. SKLDNE can effectively yield an optimal embedding subspace that finds substantial submanifolds-based structure with lower dimensionality. The within-class local structure, which is very important for face recognition, can be preserved simultaneously in a nonlinear kernel feature space. SKLDNE can solve the “out-of-sample” problem and the “overlearning of locality” problem in manifold learning, which other aforementioned methods often face. Therefore, based on these characteristics, it can be concluded that the recommended SKLDNE technique is a promising technique to be used for dimensionality reduction, with a very satisfactory performance in classification when dealing with high-dimensional data.

### 4.2. Experimental Results with the Yale Face Database

**Figure 4:**
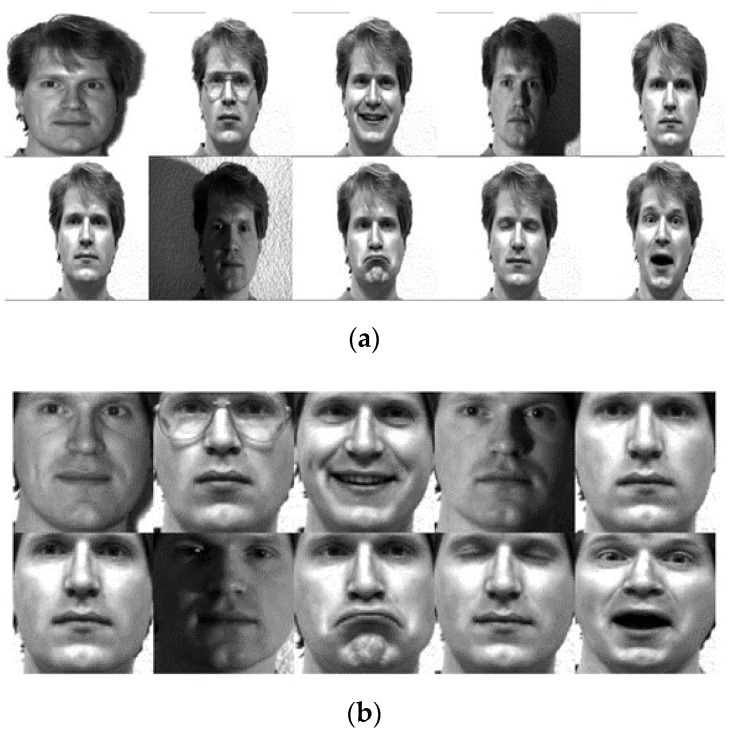
(**a**) A subset of the original Yale database. (**b**) A subset of cropped images [53].

The Yale face database [53] consists of 165 images (grayscale) taken from 15 people [54] under diverse facial expressions and light conditions. The data include 11 images for every individual, with various facial expressions or environmental conditions, such as: normal, wearing glasses and no glasses, center-light, left and right light, happy, winking, sleepy, surprised, and sad. In the experimental results, every image was first cropped and then was resized to 32 × 32 pixels [55]. Figure 4a,b illustrates some sample images of one individual of the Yale database and corresponding cropped images.

PCA was used in all methods for feature extraction, and all methods include a PCA phase. The optimum rate of recognition of each technique and the equivalent dimension implemented in the Yale database are illustrated in Table 2. Furthermore, in all experiments, due to the large quantity of implementations, it was decided to select some training and testing images that were more challenging for the classification task to clarify the performance of the aforementioned dimensionality reduction techniques in these critical areas. Considering the small training sample size problem (SSS), we first selected a training number of 1 sample and then some larger numbers from 6 to 9 to evaluate the performance of our SKLDNE method in some common existing problems such as the SSS problem and the out-of-sample problem.

**Figure 5:**
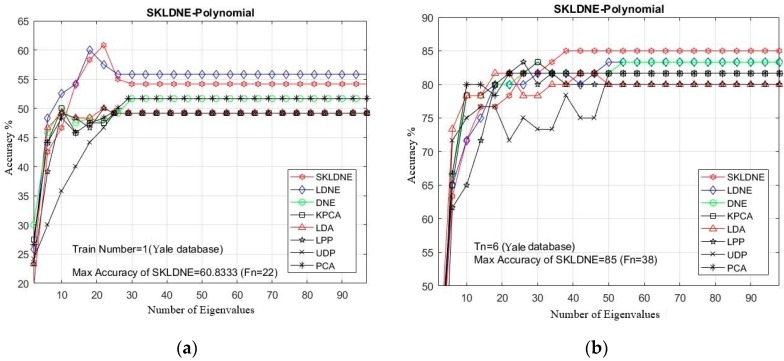

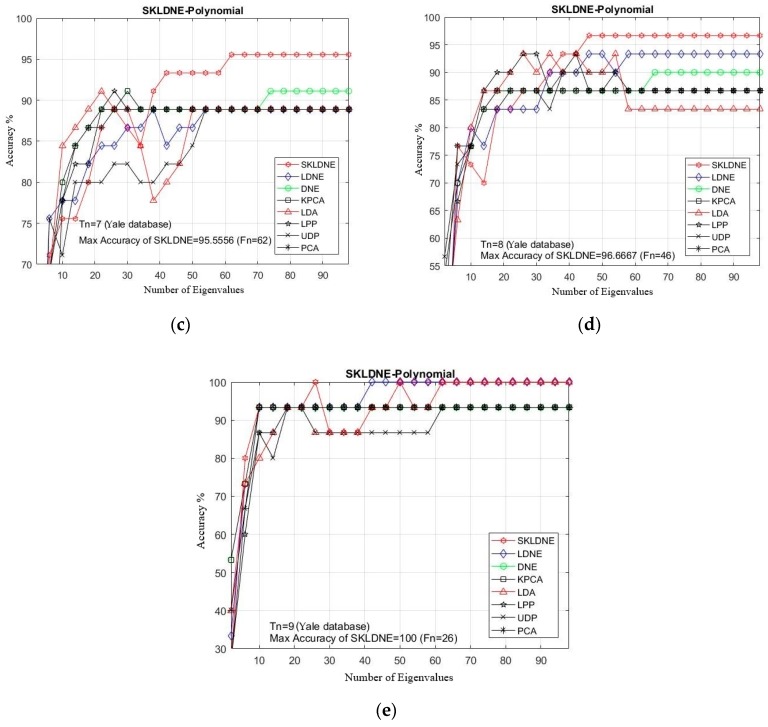
(**a**–**e**). The comparative recognition results changing the dimensionality of the transformation matrix for each given training number Tn on each dataset (Yale Face database).

Table 2 shows that the SKLDNE method achieved the highest accuracy in 100% of implementations in the Yale Database. In Figure 5a–e, the comparative classification accuracies are plotted for each given Tn (training number) in each dataset through changing the dimensionality of the transformation matrix. As is shown, the recognition rate of each technique increased promptly till the dimensionality was almost 40, and then it stabilized. It can be observed in Table 2 that SKLDNE was implemented more efficiently than others among a wide variety of dimensionality in the Yale Face database. Meanwhile, the best implementation of SKLDNE was achieved at a smaller dimension value in most of training numbers for each data set compared to LDNE. Moreover, differences in the classification between SKLDNE and other methods are very clear, especially when training number was small, for instance, *Tn* = 1, 2. For training number 7, SKLDNE yielded an improvement of around 4.5% compared with DNE, LPP, LDA, and KPCA, and 6.6% in comparison with LDNE, UDP, and PCA respectively. For training number 9, both SKLDNE and LDNE gained 100% accuracy, while accuracies in other methods with the same training number were much lower. In order to explain the superiority of the proposed method, our SKLDNE first mapped the data in the kernel space to capture the substantial extracted data and then both geometrical and discriminant information of the data were taken, benefiting from a significant form of the affinity weight matrix to embed the graph. Although LPP, DNE, and LDNE outperforming PCA, KPCA, and UDP demonstrates that the discriminant and local data structure based methods are more suitable for face recognition, our SKLDNE had more nonlinear data representation, locality preservation, and discriminating power than other methods, and consequently achieved the best recognition accuracy. Therefore, based on the mentioned characteristics of SKLDNE, it can be concluded that our SKLDNE can overcome the “SSS,” “out-of-sample,” and “overlearning” problems.

### 4.3. Experimental Results with the ORL Database

**Figure 6:**
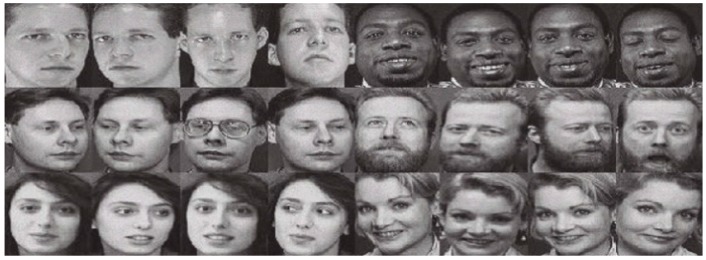
Example of six different subjects (each with 4 images) from the ORL database [56].

The ORL face database [56] includes ten diverse grayscale images taken from 40 different individuals [57]. All 10 face images of each subject were captured at different times, with changes in the lighting, facial details (with glasses or no glasses) or facial expressions (smiling/not smiling, open/closed eyes,), against a dark homogeneous background, and with straight and frontal views. The size of all images is equal to 92 × 112 pixels in PGM format. The images were captured in 40 directories [58]. It should be noted that preprocessing was used and all original images were already cropped and resized. In this project, the size of 32 × 32 pixels was chosen for all ORL images. Figure 6 illustrates three different subjects (each with 10 images) from the ORL database.

**Figure 7:**
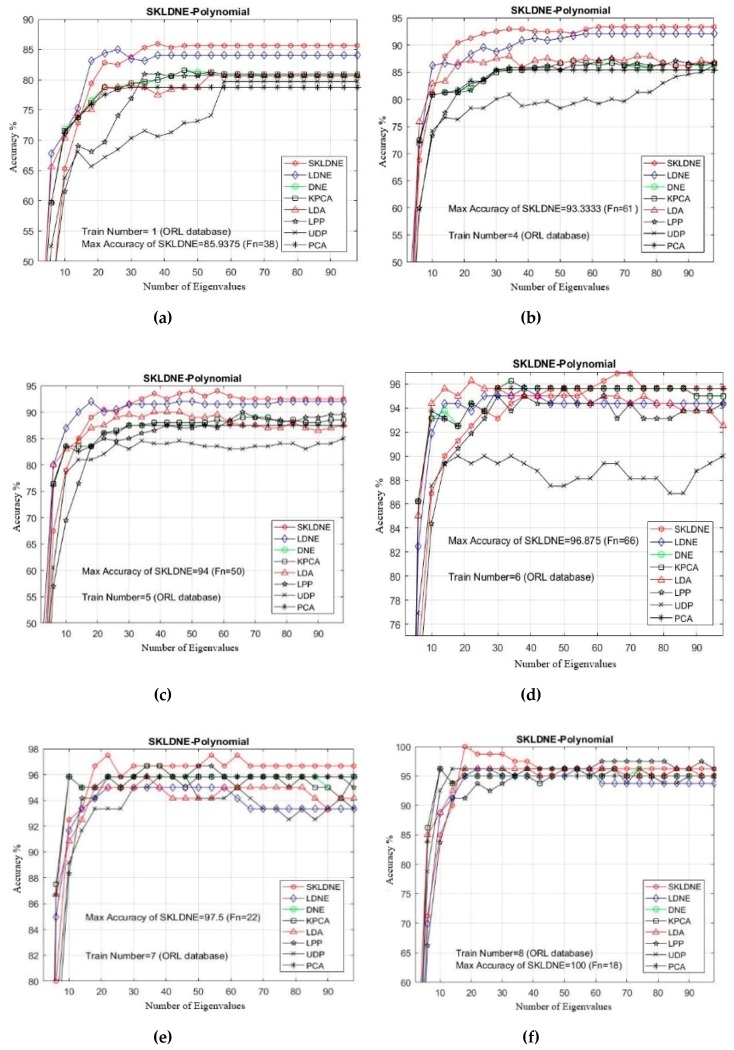
(**a**–**f**) The comparative recognition results changing the dimensionality of the transforming matrix for each given training number Tn on each data (ORL database).

In our experiments, the number of training samples *Tn* = 1, 4, 3, 4, 5, 6, 7, 8 were chosen from the dataset related to each subject to make the training sample set. The other numbers of images are applied as a testing sample set. As already mentioned, PCA was used in the classification phase in all methods. The maximal average accuracy (in percentage terms) and its corresponding dimension, followed by the alteration in the training sample sizes, are illustrated in Table 3. It can be observed in this yable and in Figure 7a–f that SKLDNE generally outperformed LDNE, whether the number of training sample size was small or not, in almost smaller numbers of dimensions. Moreover, as a supervised method, SKLDNE also significantly outperformed other techniques (KPCA, LPP, DNE, UDP, and LDA) regardless of the change in the training sample size. Compared to other techniques, SKLDNE performed better in small training sample size case. Furthermore, when the training number was equal to 8, SKLDNE had a zero error rate compared to that of PCA (4.1%), KPCA (3.5%), UDP (4%), LPP (3.5%), LDA (4%), DNE (3.75), and LDNE (3.5%). SKLDNE can simultaneously discover interclass and intraclass geometrical information, and have more nonlinear data representation, locality preservation, and discriminating power than other techniques. Therefore, SKLDNE does have merit over other techniques in terms of resolving classification problems in face recognition. This characteristic of SKLDNE in small sample size cases is indeed important to improve the recognition rate in practice, since face recognition is commonly a small sample size problem. Normally, a small number of images of each person is accessible in many real-world tasks.

### 4.4. Experimental Results with the Head Pose Database

**Figure 8:**
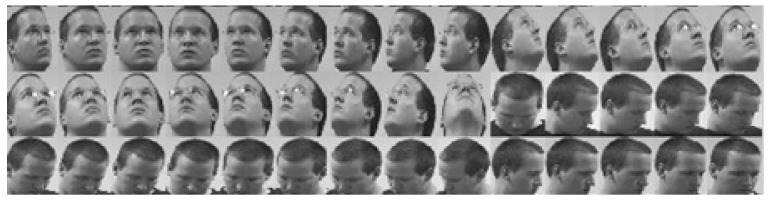
A subset of some images of one subject from the Head Pose database [59].

The Head Pose database (Figure 8) [59] includes 2790 images of faces taken from 15 individuals with pan and tilt variation angles from −90 to +90 degrees. For each individual, 2 sets with 93 different positions were captured [60].

**Figure 9:**
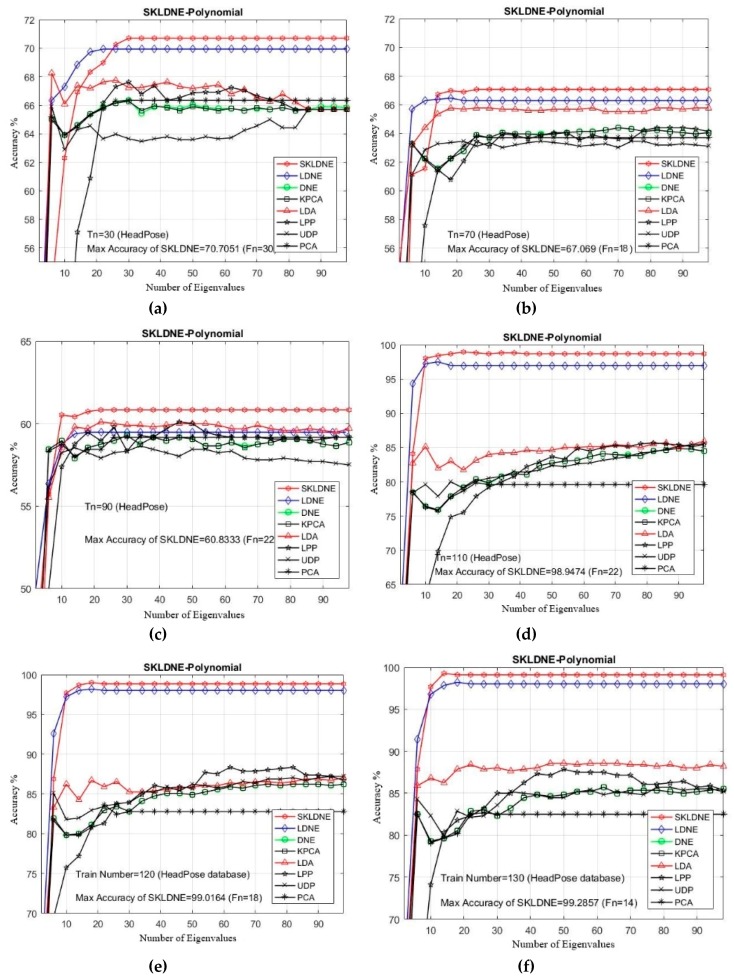
(**a**–**f**) Comparative recognition results changing the dimensionality of the transformation matrix for each given training number Tn in each dataset (Head Pose database).

As can be observed in Table 4, SKLDNE performed better than the other seven methods, regardless of the variation in the training sample size. The maximal recognition rate of SKLDNE when *Tn* = 130 was up to 99.28%, while for other methods it was much lower. This reveals that, when the given training sample size for each class gets larger, SKLDNE can obtain much better results than other methods. Two more points can also be outlined. First, our supervised method with kernel weighting can notably enhance the class classification performance, but applying the kernel trick has no significant influence on PCA performance. Second, SKLDNE achieves optimal recognition rates at almost smaller number of dimensions as the recognition rate of SKLDNE retains the best results as the dimension varies from 14 to 30. Compared to the other techniques, SKLDNE preserves the more discriminative and local features of face images. It also preserves more local geometric relations of the within-class samples by nonlinear kernel mapping. It should be noted that linear methods such as LPP, LDA, LDNE, DNE, and UDP often fail to deliver good classification performance when face images are subject to complex nonlinear changes such as expression, lighting, pose and so on. Figure 9 indicates that the recognition implementations of all methods first sharply increase while the projected dimensions are added, and then, after obtaining the optimum, they tend to become stable.

### 4.5. Experimental Results with the Finger Vein Database.

**Figure 10:**
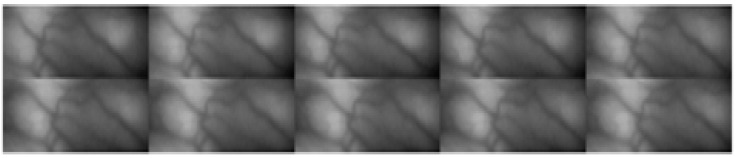
Example of captured images of one person in the Finger Vein database [61].

The finger vein database used in this project was collected from 51 individuals (male and female) who were aged between 21 and 56 [61]. 10 images were captured from each subject. Four fingers were used for capturing, including right and left middle finger and right and left index finger. There are 204 different fingers in the database, and the data consist of 2040 images in total, in which each finger image originally had a dimension of 480 × 160 pixels. In our implementations, each image was resized to 32 × 32. The captured images from one person can be seen in Figure 10.

In this section, the performance of each technique followed by a maximal recognition rate was evaluated through changing the dimensionality of transformation matrix. The number of training samples *Tn* = 1, 2, 3, 4, 5, 6, 7 was chosen from the image gallery to make the training sample set, and the rest of images were selected for testing [62]. Furthermore, the PCA classifier (nearest neighbor NN using the Euclidean distance) was applied in the recognition phase, and the best recognition rates of each technique are indicated in Table 5.

**Figure 11:**
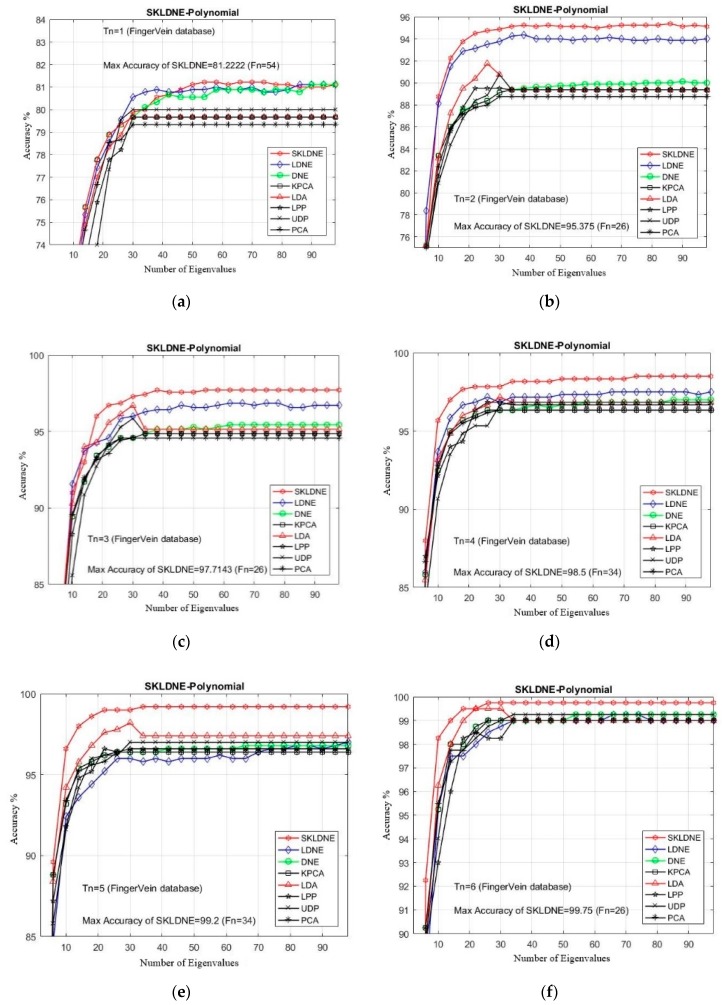

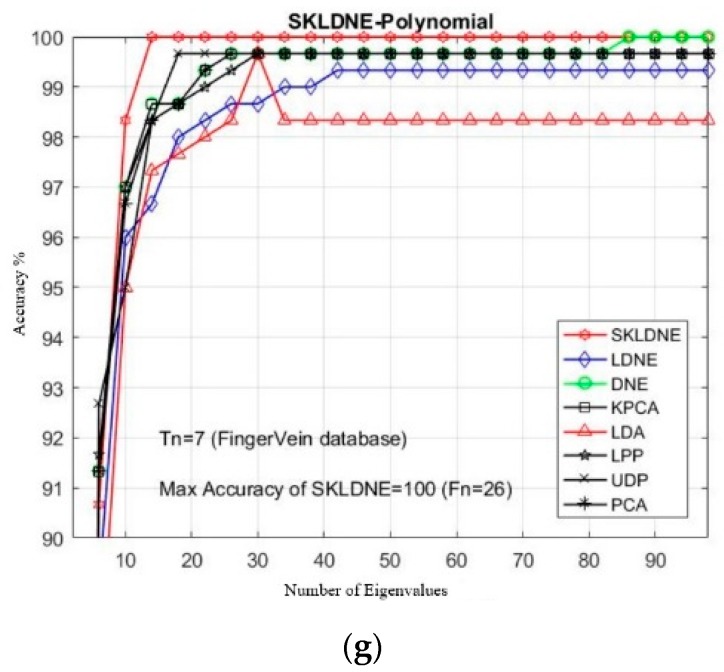
(**a**–**g**). Comparative recognition results changing the dimensionality of the transformation matrix for each given training number *Tn* (Finger Vein database).

Based on the experiment results shown in Figure 11, our SKLDNE gained the best recognition rate among all different training numbers, which proves that it has an encouraging performance compared to other advanced methods. Regarding the small training sample size case, the SKLDNE method still showed that it performed significantly better than other techniques, as the maximal recognition accuracy rate of SKLDNE in training number 2 was almost 5% more than PCA, KPCA, UDP, LPP, and DNE, and 1.2% more than LDNE. Our SKLDNE was always able to represent its optimal embedding space with a lower value of dimensions in comparison with the other seven techniques. For example, SKLDNE for *Tn* = 7 achieved 100% recognition accuracy in the smallest value of projected dimensions (26). This conveys that our approach is more effective, due to its significant characteristic that it can not only represent nonlinear and complex variations in of images, but can also model both locality of LPP and discrimination of DNE simultaneously. This fact demonstrates the good performance of our proposed method.

### 4.6. Experimental Results with the Finger Knuckle Print Database

**Figure 12:**
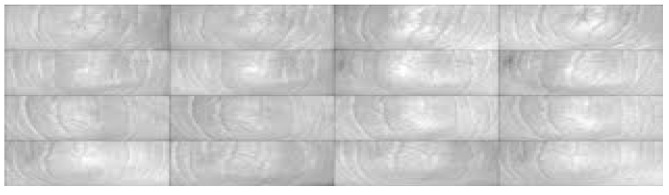
A cropped sample of the finger knuckle print (FKP) database [63].

This database is organized by the Polytechnic University of Hong Kong [63], and is freely available online. Based on the database description, finger knuckle print (FKP) images were collected from 165 individual volunteers (males and females) [64]. The samples were collected in two distinct sessions, and in each one 6 images were captured from 4 fingers (including left and right index finger and the left and right middle finger). Therefore, 7920 finger images in total were taken from 660 various fingers. In our experiments, the original image of the database is cropped and is then resized to 32 × 32 pixels. Figure 12 illustrates a cropped sample of the FKP database.

**Figure 13:**
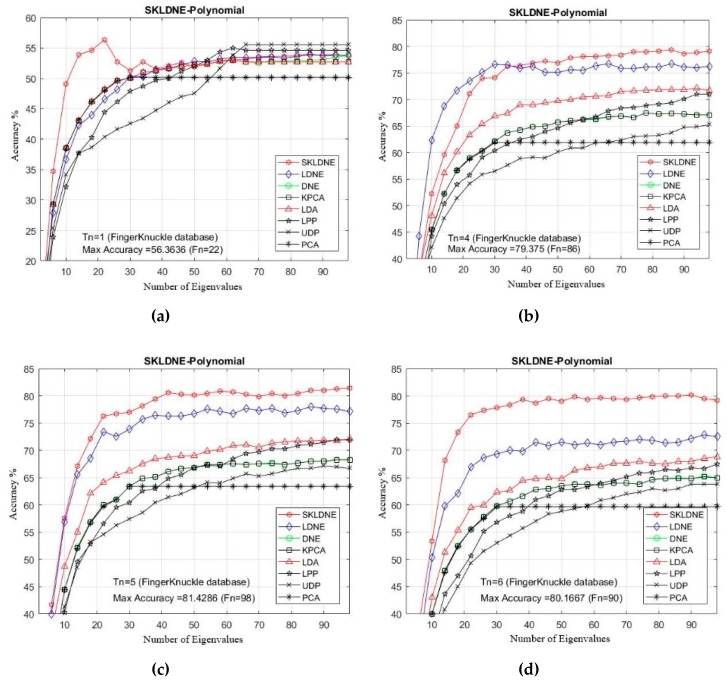

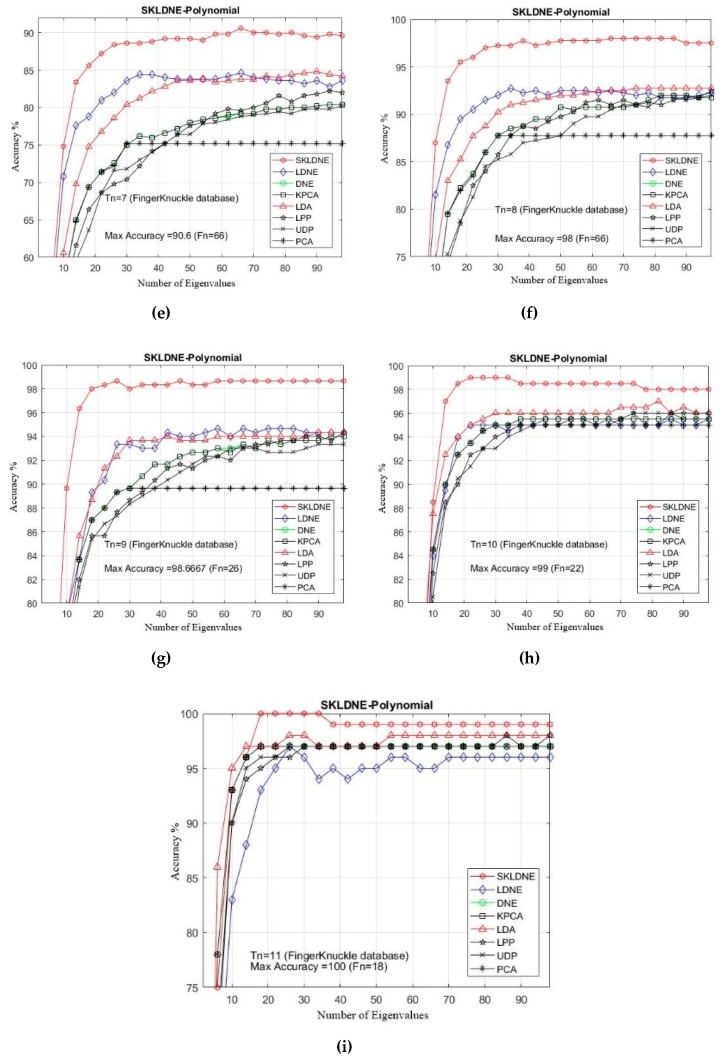
(**a**–**i**). Comparative recognition results changing the dimensionality of the transformation matrix for each given training number Tn (Finger Knuckle database).

Table 6 shows that the SKLDNE recognition performance was significantly more efficient than other techniques, regardless of the variation in the training sample size, in the Finger Knuckle database. The recognition rate of SKLDNE when *Tn* = 11 was equal to 100%, while for the LDNE it was equal to 97.2%. Another point that is worth mentioning on the comparison of recognition performance in SKLDNE and other methods is related to the small training sample size case, as SKLDNE had the best performance in this respect. Again, all best recognition performances of SKLDNE were almost achieved at smaller values of dimension on every Tn per data set. It can also be observed that, when the given training sample size of each class became larger, SKLDNE achieved much better results than other techniques. For example, for *Tn* = 6, the accuracy of SKLDNE was around 8% and 21% more than LDNE and PCA, respectively. In order to well explain the superiority of SKLDNE, it should be noted that it can preserve more effective nonlinear features and more geometrical and discriminant information so, it can tackle the small sample size, the out-of-sample, and the “overlearning of locality” problems. Hence, it can be concluded that the SKLDNE approach is a promising supervised technique with a satisfactory performance of classification in order to be applied in the Finger Knuckle database. The recognition rates in comparison with the variation of dimensions for each Tn are shown in Figure 13a–i.

### 4.7. Classification Performance and Computational Cost

**Figure 14:**
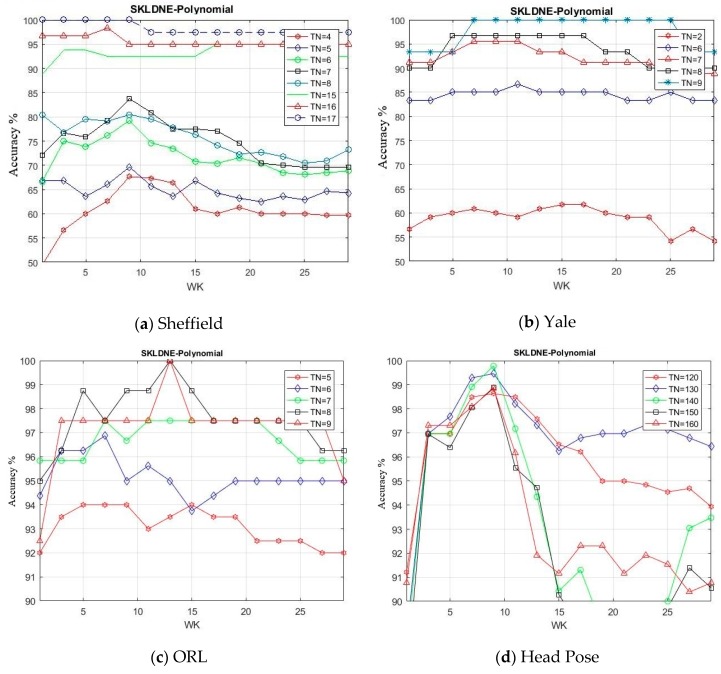

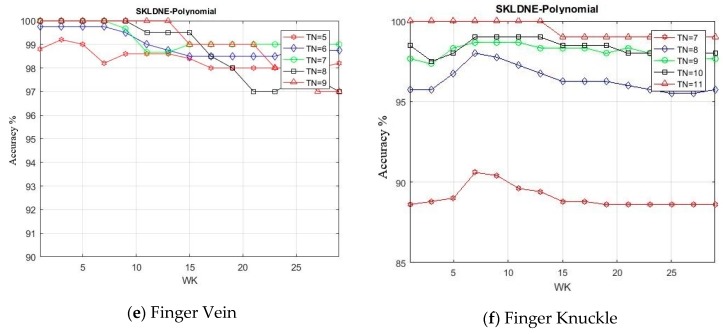
(**a**–**f**). Maximum recognition rate of SKLDNE versus Wk for different number of training samples on Sheffield, Yale, ORL, Head Pose, Finger Vein and Finger Knuckle databases.

In this section, by means of a new group of experiments, we evaluated SKLDNE performance through changing k-neighborhood variation *Wk* (from 1 to 30 with a scale of 2). The number of training samples selected for each database were *Tn* = 4, 5, 6, 7, 8, 15, 16, 17 in Sheffield, *Tn* = 2, 6, 7, 8, 9 in Yale, *Tn* = 5, 6, 7, 8, 9 in ORL, *Tn* = 120, 130, 140, 150, 160 in Head Pose, *Tn* = 5, 6, 7, 8, 9 in Finger Vein, and *Tn* = 7, 8, 9, 10, 11 in Finger Knuckle databases. It should be noted that the rest of each class in each dataset was used for testing. The training number samples were randomly selected. The maximum recognition rates of SKLDNE in comparison with *Wk* for different numbers of training samples are indicated in Figure 14a–f.

It can be seen that the classification performance became better as the number of training samples increased. The classification performance of SKLDNE improved first with a rise of *Wk* until almost *Wk* = 9 in the Sheffield and Head Pose, and then it decreased dramatically. In the Yale, ORL, Finger Vein, and Finger Knuckle datasets, it can easily be observed that the recognition performance of SKLDNE enhanced rapidly when *Wk* varied from 1 to 7 in Yale, 1 to 10 in ORL, 1 to 9 in Finger Vein, from 1 to 6 in Finger Knuckle, and then it decreases when *Wk* became larger, since large values of k-neighborhood variable *Wk* have an effect on creating the weight adjacent matrix. It has already been proven that the k-neighborhood selected for data point might contain more outliers belonging to other classes at a large number of *Wk* when a dataset includes many classes with a small number of samples for each class. Thus, the constructed adjacent weight matrix does not have sufficient discrimination for image recognition [15].

The experiment for analyzing computational cost was carried out on an Intel(R) Core i5-4200U CPU, 2.3 GHz, 10 GB RAM machine using MATLAB (R2016, Natick, MA, USA). The computational costs of the different classification methods using the Yale data base are listed in Table 7.

From the results shown in Table 7, it can be observed that the proposed SKLDNE was faster than its main competitors, such as LDNE, DNE, and LPP. The processing times of PCA, KPCA, UDP, and LDA were lower. However, the recognition rate results illustrate that these methods were much less accurate than the SKLDNE method.

Now, to have a more reliable comparison, we briefly compare our recognition results of the proposed method with previously published works, including a deep learning method named Deep Belief Networks (DBNs) [65,66] with a traditional multilayer perceptron model (MLP) [66] in the used facial expression databases, i.e., the JAFFE Database [67]. As a deep learning method, DBNs have an unsupervised feature learning ability. The JAFFE database includes 10 individuals (Japanese women) with 7 different expressions, and has around 3 or 4 images for each expression. There are 213 images in total in this database. Each image has a resolution pixel of 256 × 256. In detail, we divided all image samples into 10 parts, 90% of which were applied for training, and the remaining were applied for testing. Table 8 illustrates the recognition performance comparisons in the JAFFE database when dealing with three different image resolutions of 16 × 16, 32 × 32, and 64 × 64. We can clearly see that the proposed SKLDNE method achieved the best recognition performance (100% in all cases), in comparison with other previously reported results [66], which are much lower. This is attributed to the main characteristics of SKLDNE, which effectively represents more nonlinear data structure and has more locality and discrimination information preserving power. The results again show the robustness of SKLDNE for facial expression recognition.

## 5. Conclusions and Future Research

In this study, the performance of several well-known pattern recognition strategies was analyzed to clarify which techniques are best suited to be applied in face recognition. We also analyzed the weakness and robustness of each technique. As already mentioned, DNE cannot correctly preserve local information of data because it only assigns +1 to intra-class and −1 to inter-class neighbors, so it might fail to discover the most significant submanifolds for pattern classification. LPP is designed based on “locality” since it has no direct connection with classification, and it still suffers from the “over learning of locality” problem. LDNE has been proposed to overcome the problems existing in LPP and DNE; however, it does not guarantee an appropriate projection for classification purposes because many important non-linear data might be lost during its dimensionality reduction process. In addition, in some cases, LDNE cannot distinguish inter-class and intra-class neighbors well either in order to conduct projection for all points. This can degrade the classification performance. In order to address these problems, we have proposed a new supervised subspace learning algorithm named “supervised kernel locality-based discriminant neighborhood embedding” (SKLDNE). Combined with nonlinear data structures, locality, and discrimination information, SKLDNE can yield an optimal subspace that best finds the indispensable submanifolds-based structure. Six publicly available datasets, i.e., Yale face, ORL face, Sheffield, Head Pose, Finger Vein and Finger Knuckle, were used to illustrate the significance of the proposed technique. Experimental results reveal that SKLDNE outperforms and demonstrates potential to be implemented in real world systems compared to other advanced dimensionality reduction methods by obtaining the highest recognition rates in all experiments. Representing complex nonlinear variations makes SKLDNE more powerful and more intuitive than LDNE and other aforementioned techniques in terms of classification. According to the results, SKLDNE could also resolve the “small training sample size” problem, and it had the best performance compared to others at smaller numbers of projected dimensions in each number of training samples per data set. Moreover, when the given training sample size for each class grew larger, SKLDNE also achieved much better results than other techniques. The overlearning of locality problem and the out-of-sample problem in manifold learning can be avoided by applying our developed classifier. As a future plan, we will modify this classifier in order to make it directly applicable for two-dimensional data to effectively reduce the computational costs.

## Figures and Tables

**Table 1:** Maximum recognition accuracies (in percentage terms) of supervised kernel locality-based discriminant neighborhood embedding (SKLDNE) and other methods for the different numbers of training and testing images on the Sheffield Face and corresponding dimensions (shown in parentheses).

Database	Sheffield Face										
**Tn**	**1**	**2**	**3**	**4**	**5**	**6**	**7**	**8**	**15**	**16**	**17**
**PCA**	45.1	47.94	49.68	49	49.64	51.15	52.5	54.54	85.5	93	92
	(18)	(18)	(18)	(18)	(30)	(26)	(30)	(26)	(18)	(28)	(10)
**KPCA**	45.2	48.2	50.31	50.33	50.7	51.92	54.15	55.9	87.5	93	92
	(18)	(22)	(30)	(38)	(42)	(58)	(50)	(69)	(38)	(30)	(14)
**UDP**	45.33	48.1	48.43	51.66	50.71	51.9	55.41	57.27	87	95	92.5
	(10)	(22)	(30)	(30)	(45)	(66)	(54)	(50)	(90)	(86)	(14)
**LPP**	45.55	48.2	52.81	50.66	56.07	52.3	54.16	55.9	90	93.33	95
	(14)	(26)	(12)	(34)	(38)	(54)	(46)	(62)	(74)	(42)	(34)
**LDA**	45.2	50.29	48.43	58.66	56.07	59.23	60	61.36	92.5	93	97.5
	(18)	(26)	(14)	(34)	(22)	(6)	(42)	(50)	(22)	(30)	(18)
**DNE**	45.2	48.23	50.31	51.33	51.78	51.9	54.58	56.36	87.5	93.33	92.5
	(18)	(34)	(14)	(74)	(58)	(42)	(74)	(78)	(38)	(30)	(20)
**LDNE**	45.27	50.58	56.87	58.66	65	73.07	76.66	76.81	90	96.1	97.5
	(22)	(9)	(14)	(14)	(14)	(34)	(18)	(14)	(22)	(14)	(18)
**SKLDNE**	**46.38**	**52.94**	**59.06**	**62.66**	**69.64**	**78.46**	**83.75**	**80.45**	**93.75**	**98.33**	**100**
	(10)	(10)	(14)	(10)	(14)	(30)	(10)	(10)	(34)	(10)	(10)

**Table 2:** Maximum recognition accuracies (in percentage terms) of SKLDNE and other methods for the different number of training and testing images on Yale Face database and corresponding dimensions (shown in parentheses).

Database	Yale Face				
Tn	1	6	7	8	9
PCA	51.66	81.66	88.88	86.6	93
	(29)	(22)	(26)	(26)	(10)
KPCA	50	83.3	91	86.66	93.3
	(10)	(30)	(30)	(90)	(10)
UDP	49.16	81.66	88.8	90	92.9
	(25)	(50)	(54)	(28)	(18)
LPP	51	85	91.1	96.66	93.3
	(22)	(26)	(30)	(34)	(18)
LDA	50	81.66	91.1	93.3	93.3
	(22)	(18)	(22)	(98)	(50)
DNE	51.66	83.3	91	90	93
	(30)	(30)	(30)	(66)	(10)
LDNE	60	83.3	88.88	93.33	**100**
	(19)	(50)	(57)	(48)	(42)
SKLDNE	**60.83**	**85**	**95.55**	**96.66**	**100**
	(22)	(38)	(62)	(46)	(26)

**Table 3:** Maximum recognition accuracies (in percentage terms) of SKLDNE and other methods for the different number of training and testing images in the ORL Face database and corresponding dimensions (shown in parentheses).

Database	ORL Face					
Tn	**1**	**4**	**5**	**6**	**7**	**8**
PCA	78.75	85.41	87.5	95.62	95.83	95.9
	(30)	(30)	(26)	(20)	(10)	(10)
KPCA	81.56	87	89	96.2	96.66	96.25
	(46)	(54)	(66)	(34)	(34)	(20)
UDP	80	86.66	89.5	94.75	96.6	96
	(54)	(90)	(98)	(38)	(18)	(14)
LPP	80.62	87.5	90	95	95.8	97.5
	(58)	(86)	(94)	(34)	(30)	(62)
LDA	80.93	87.91	90	96.25	95.83	96
	(54)	(34)	(38)	(22)	(34)	(18)
DNE	81.56	87.08	89	96.2	96.66	96.25
	(46)	(54)	(66)	(34)	(34)	(10)
LDNE	85	92	92	95.6	95	96.5
	(26)	(62)	(78)	(30)	(24)	(54)
SKLDNE	**85.93**	**93.33**	**94**	**96.87**	**97.5**	**100**
	(38)	(61)	(50)	(66)	(22)	(18)

**Table 4:** Maximum recognition accuracies (in percentage terms) of SKLDNE and other methods for the different number of training and testing images on Head Pose database and corresponding dimensions (shown in parentheses).

Database	Head Pose					
Tn	**30**	**70**	**90**	**110**	**120**	**130**
PCA	66.21	63.69	50.16	79.73	83.6	82.67
	(30)	(26)	(26)	(26)	(74)	(26)
KPCA	66.38	64.31	59.37	84.86	86.22	85.71
	(30)	(70)	(30)	(90)	(22)	(62)
UDP	65.83	64.39	58.75	85.52	87.21	85.71
	(6)	(22)	(34)	(98)	(94)	(78)
LPP	68.01	64.4	59.7	85.39	88.36	87.85
	(30)	(82)	(26)	(90)	(62)	(50)
LDA	68.21	65.14	60	86.57	87.04	88.57
	(6)	(18)	(22)	(98)	(98)	(46)
DNE	66.28	64.5	58.5	84.63	86.22	85.71
	(30)	(70)	(30)	(90)	(74)	(62)
LDNE	69.7	66.2	59	96.9	98	98.02
	(20)	(19)	(18)	(24)	(18)	(18)
SKLDNE	**70.7**	**67.06**	**60.83**	**98.94**	**99.01**	**99.28**
	(30)	(18)	(22)	(22)	(18)	(14)

**Table 5:** Maximum recognition accuracies (in percentage terms) of SKLDNE and other methods for the different number of training and testing images on Finger Vein database and corresponding dimensions (shown in parentheses).

Database	Finger Vein						
Tn	**1**	**2**	**3**	**4**	**5**	**6**	**7**
PCA	79.66	89.37	94.85	96.3	96.41	99	99.54
	(30)	(34)	(34)	(26)	(26)	(26)	(27)
KPCA	79.33	88.75	94.57	96.33	96.4	99.2	99.5
	(30)	(30)	(30)	(30)	(26)	(26)	(27)
UDP	80	90.75	95.85	96.8	97	99	99.5
	(30)	(30)	(30)	(34)	(30)	(34)	(30)
LPP	79.66	89.5	94.85	96.83	96.6	99	99.56
	(30)	(22)	(34)	(26)	(22)	(34)	(34)
LDA	79.66	91.75	96.71	97.16	98.2	99.5	99.55
	(30)	(26)	(30)	(30)	(30)	(22)	(29)
DNE	81	90.12	95.42	97	96.8	99.15	**100**
	(90)	(90)	(62)	(86)	(66)	(54)	(86)
LDNE	80.75	94.17	96.85	97.5	96.4	99.25	99.2
	(86)	(38)	(62)	(76)	(74)	(66)	(42)
SKLDNE	**81.22**	**95.38**	**97.71**	**98.5**	**99.2**	**99.75**	**100**
	(54)	(26)	(26)	(34)	(34)	(26)	(26)

**Table 6:** Maximum recognition accuracies (in percentage terms) of SKLDNE and other methods for the different number of training and testing images on Finger Knuckle database and corresponding dimensions (shown in parentheses).

Database	Finger Knuckle								
Tn	**1**	**4**	**5**	**6**	**7**	**8**	**9**	**10**	**11**
PCA	50.18	67.5	68.28	59.66	75.2	92	94	93	97.5
	(30)	(78)	(94)	(30)	(26)	(82)	(90)	(38)	(40)
KPCA	52.9	61.87	63.42	59.66	80.2	87.75	89.66	93	97.15
	(50)	(60)	(39)	(37)	(30)	(25)	(26)	(27)	(20)
UDP	56.18	65.25	67.14	63.88	82.2	92	93.3	96.5	97
	(62)	(98)	(90)	(90)	(98)	(98)	(90)	(70)	(35)
LPP	55	71	72	67.5	82.2	92.5	94.2	96	98
	(62)	(94)	(94)	(98)	(94)	(98)	(98)	(74)	(86)
LDA	53	72.12	72.14	68.83	84.8	92.7	94.33	97	98
	(62)	(94)	(98)	(98)	(90)	(74)	(90)	(82)	(26)
DNE	53.81	67.5	68.3	65.33	80.4	92	94	93	97
	(98)	(88)	(94)	(94)	(94)	(82)	(90)	(38)	(20)
LDNE	53.9	76.75	78	72.70	84.6	92.75	94.66	93.3	97.2
	(86)	(87)	(86)	(94)	(76)	(34)	(58)	(46)	(26)
SKLDNE	**56.36**	**79.37**	**81.42**	**80.16**	**90.6**	**98**	**98.66**	**99**	**100**
	(22)	(86)	(98)	(90)	(66)	(66)	(26)	(22)	(18)

**Table 7:** The computational costs of the different classification methods using the Yale data base.

Method	SKLDNE	LDNE	DNE	KPCA	LDA	LPP	UDP	PCA
Time (s)	0.35	0.4	0.37	0.012	0.04	0.36	0.06	0.02

**Table 8:** Maximum recognition accuracies (in percentage terms) of SKLDNE and other methods on the JAFFE database.

Method	16 × 16	32 × 32	64 × 64
MLP	64.76	84.76	86.19
DBNs + MLP	88.57	89.05	90.95
SKLDNE	**100**	**100**	**100**
